# Evaluation of Transport Properties and Energy Conversion of Bacterial Cellulose Membrane Using Peusner Network Thermodynamics

**DOI:** 10.3390/e25010003

**Published:** 2022-12-20

**Authors:** Izabella Ślęzak-Prochazka, Kornelia M. Batko, Andrzej Ślęzak

**Affiliations:** 1 Biotechnology Centre, Silesian University of Technology, Akademicka 2A, 44-100 Gliwice, Poland; 2 Institute of Political Science, Faculty of Social Sciences, University of Silesia, Bankowa 12, 40-007 Katowice, Poland; 3 Faculty of Health Science, Jan Dlugosz University, 13/15 Armia Krajowa Al, 42-200 Częstochowa, Poland

**Keywords:** membrane transport, Kedem–Katchalsky–Peusner equations, bacterial cellulose membrane, concentration polarization, Peusner coefficients of membranes, energy conversion

## Abstract

We evaluated the transport properties of a bacterial cellulose (BC) membrane for aqueous ethanol solutions. Using the *R^r^* version of the Kedem–Katchalsky–Peusner formalism (KKP) for the concentration polarization (CP) conditions of solutions, the osmotic and diffusion fluxes as well as the membrane transport parameters were determined, such as the hydraulic permeability (*L_p_*), reflection (σ), and solute permeability (ω). We used these parameters and the Peusner ($R_{ij}^{r}$) coefficients resulting from the KKP equations to assess the transport properties of the membrane based on the calculated dependence of the concentration coefficients: the resistance, coupling, and energy conversion efficiency for aqueous ethanol solutions. The transport properties of the membrane depended on the hydrodynamic conditions of the osmotic diffusion transport. The resistance coefficients $R_{11}^{r}$, $R_{22}^{r}$, and $R_{det}^{r}$ were positive and higher, and the $R_{12}^{r}$ coefficient was negative and lower under CP conditions (higher in convective than nonconvective states). The energy conversion was evaluated and fluxes were calculated for the *U*-, *F*-, and *S*-energy. It was found that the energy conversion was greater and the *S*-energy and *F*-energy were lower under CP conditions. The convection effect was negative, which means that convection movements were directed vertically upwards. Understanding the membrane transport properties and mechanisms could help to develop and improve the membrane technologies and techniques used in medicine and in water and wastewater treatment processes.

## 1. Introduction

Membranes exert multiple functions, including protective, regulatory, and coordinating functions [1]. The protective and regulatory functions are based on the membrane selectivity of a barrier that regulates the transport between the interior of a system and its surroundings [2]. The separation properties of synthetic polymer membranes enable their application in many fields of science, technology, and medicine, such as food production, water treatment, hemodialysis, wastewater treatment, and membrane dressings [1,3]. The coordinating function seems to apply only to biological systems, where the membrane simultaneously plays the role of a receiver, regulator, and coordinator of environmental signals, which are the driving forces of membrane transport [4]. These driving forces cause various types of physical fields, such as concentration, pressure, temperature, or electric potentials, which participate in shaping the field character of nature [5,6].

Selective permeability is one of the basic properties of porous media, including polymeric membrane-forming materials. It is required for large-scale models of fluid flow and mass transport. These models operate within the framework of nonequilibrium thermodynamics, hydrodynamics, and statistical physics [7]. The last 20 years have seen a significant increase in the use of modeling to study multiphase flow and transport in porous media. Starting with models of fluid systems in single pores, calculations of the relative permeability, interfacial area, dissolution rate, and many other physical properties have been carried out [8]. One of the more interesting methods for pore-scale numerical studies is direct hydrodynamic simulation (DHD) technology [9], which uses a description of the dispersed interface and is applied to various fluid–rock or fluid–fluid interactions for equal rheological conditions.

In the paper [10], a stochastic method based on simulated annealing and X-ray microtomography was used to study the pore structures of various porous solids that differ in pore space morphology and topology. In the process of verifying the developed models, many interesting simulation and experimental results were obtained, which confirmed the pore space models. In addition, it was shown that predictions based on tomographic pore space models were more effective than stochastic models and that the time-dependent effective diffusivity is particularly sensitive to small morphological deviations between the actual and reconstructed pore structures. It was also shown that the combined prediction of the effective permeability, effective pore size, geometric coefficient, and time-dependent effective water diffusivity is needed to reliably evaluate pore space reconstruction.

The paper [11] presented a description of volume membrane transport using the Kedem–Katchalsky equations of homogeneous aqueous solutions of ethanol and glucose. The flows generated by the hydrostatic pressure differential, the osmotic pressure differential, and the simultaneous action of these two thermodynamic drives were analyzed independently. In addition, a formula for the membrane filtration coefficient was presented, taking into account the density and viscosity of ethanol, and the corresponding calculations were made. In this way, it was shown that the membrane filtration coefficient depends on both the membrane properties and the flowing fluid. In turn, the paper [12] presented formulas for calculating the viscosity and diffusion coefficients of binary aqueous nonelectrolyte solutions as a function of the solution concentration under isothermal conditions. In the process of verifying the obtained formulas, the dependence of the diffusion coefficient, filtration coefficient, and dynamic viscosity coefficient for these solutions on the solution concentration was calculated. Based on the obtained formulas, the results of calculations of the diffusion coefficient, membrane filtration coefficient, and dynamic viscosity of aqueous solutions of ethanol and glucose were presented.

Processes such as diffusion or osmosis can modify physical fields, including concentration fields. In addition, a concentration field can be modified by the concentration polarization (CP) as a consequence of the creation of the concentration boundary layers (CBLs) $l_{h}^{r}$ and $l_{l}^{r}$ on both sides of the membrane [5,13]. The thickness of CBL $l_{h}^{r}$ is $\delta_{h}^{r}$, and the thickness of CBL $l_{l}^{r}$ is $\delta_{l}^{r}$. As a consequence of the CBL formation, the concentration difference decreases from the value of $C_{h\;}$–$\;C_{l}$ to the value of $C_{h}^{r}-C_{l}^{r}$, where $C_{h}^{r}$ > $C_{l}^{r}$, $C_{h}$ > $C_{h}^{r}$, and $C_{l}^{r}$ > $C_{l}$, and the density difference increases from $\rho_{h}$ − $\rho_{l}$ to the value of $\rho_{h}^{r}-\rho_{l}^{r}$, where $\rho_{h}^{r}$ > $\rho_{l}^{r}$, $\rho_{h}$ > $\rho_{h}^{r}$, and $\rho_{l}^{r}$ > $\rho_{l}$. When a lower density solution is placed in the compartment under the membrane and a higher density solution is placed in the compartment above the membrane, the complex $l_{h}^{r}$/M/$l_{l}^{r}$ loses its hydrodynamic stability. Hydrodynamic instability is manifested by natural convection in near-membrane areas [13,14,15].

Then, the concentration Rayleigh number ($R_{C}$), which controls the process of the appearance of gravitational convection, exceeds its critical value, and hydrodynamic instabilities appear in the near-membrane areas [5,14,16,17,18]. Over time, the destructive effect of gravitational convection limits the growth of $\delta_{h}^{r}$ and $\delta_{l}^{r}$ and accelerates the diffusion of substances beyond the layers, which extends the effect of convection to the entire volume of the solution. Under certain conditions, even self-organization of the liquid may occur, which is manifested in the ”plum structure” [19]. The creation of CBLs can be visualized by a Mach–Zehnder laser interferometer [14]. The consequence of CP is a significant reduction in concentration gradients, as evidenced by the minimization of the osmotic and diffusion fluxes of dissolved substances and the membrane potentials [6,13,15]. Under certain conditions, depending on the compositions of the solutions and the orientation of the artificial biomembrane in relation to the gravity vector, concentration gradients can be reconstructed by gravitational convection [6,13,20]. The basic research tools for describing membrane transport are the Kedem–Katchalsky (KK) equations, both the classical version [3,21] and the modified forms [13,16,22]. For the concentration polarization conditions of the solutions, the equations have the form

(1) $$J_{v}^{r}=\zeta_{p}^{r}L_{p}\left(∆P-\zeta_{v}^{r}\sigma RT∆C\right)$$

(2) $$J_{s}^{r}=\zeta_{s}^{r}\omega RT∆C+\bar{C}\left(1-\zeta_{a}^{r}\sigma \right)J_{v}^{r}$$

where $L_{P}$, σ, and ω are the hydraulic permeability, reflection, and solute permeability coefficients; ΔP = $P_{h}$ − $P_{l}$ and Δπ = RTΔC are the hydrostatic and osmotic pressure differences (RT is the product of the gas constant and temperature, ΔC = $C_{h}$ − $C_{l}$, $C_{h}$ and $C_{l}$ are the solutes concentrations, and ΔC is the solution concentration difference); $J_{v}^{r}$ and $J_{s}^{r}$ are the volume and solute fluxes; *σ* is the reflection coefficient; ω is the permeability coefficient of the solute; $\bar{C}$ = ($C_{h}$ − $C_{l}$)[ln ($C_{h}C_{l}$^−1^)]^−1^ is the average concentration of the solutes; and $\zeta_{p}^{r}$, $\zeta_{v}^{r}$, $\zeta_{s}^{r}$, and $\zeta_{a}^{r}$ are, respectively, the hydraulic, osmotic, diffusive, and advective coefficients of the CP [23]. For dilute nonelectrolyte solutions, $\sigma_{v}$ = $\sigma_{s}$. In contrast, for nondilute solutions, $\sigma_{v}$ ≠ $\sigma_{s}$ [16].

From Equations (1) and (2), the phenomenological coefficients of homogeneous solutions ($\zeta_{p}^{r}$ = $\zeta_{v}^{r}$ = $\zeta_{s}^{r}$ = $\zeta_{a}^{r}$ = 1) are defined as follows:

(2a) $$L_{p}={\left.\frac{J_{v}}{∆P}\right|}_{∆C=0}$$

(2b) $$\sigma_{v}={\left.\frac{∆P}{RT∆C}\right|}_{J_{v}=0}$$

(2c) $$\sigma_{s}={\left.1-\frac{J_{s}}{\bar{C}J_{v}}\right|}_{∆C=0}$$

(2d) $$\omega ={\left.\frac{J_{s}}{RT∆C}\right|}_{J_{v}=0}$$

The tetrad of membrane transport parameters ($L_{P}$, $\sigma_{v}$, $\sigma_{s}$, and ω) play the roles of proportionality coefficients. The coefficients $\zeta_{p}^{r}$, $\zeta_{v}^{r}$, $\zeta_{s}^{r},$ and $\zeta_{a}^{r}$ play similar roles. The products $L_{P}\zeta_{p}^{r}$, $\sigma \zeta_{v}^{r}$, $\omega \zeta_{s}^{r},$ and $\sigma \zeta_{a}^{r}$ determine the transport properties of the membrane complex and the concentration boundary layers.

The values of the $L_{P}$, σ, and ω coefficients, for isotropic and electrically neutral artificial membranes and for dilute solutions, are constant. Examples of such membranes are those made of regenerated cellulose (Nephprophan and Cuprophan) and bacterial cellulose (Biofill) [24,25,26]. The values of these coefficients for compound and ion-exchange membranes (Nafion and Textus bioactiv) are concentration-dependent [27,28,29,30].

The Kedem–Katchalsky–Peusner (KKP) equations are the network forms of the KK equations proposed by L. Peusner, which are obtained by means of the symmetrical or hybrid transformations proposed by Peusner network thermodynamics [25]. These equations contain the Peusner coefficients ($R_{ij}$), which for the conditions of homogeneity of solutions are a combination of the phenomenological coefficients of the membrane (*L_p_*, *σ*, and *ω*) and the average concentration of the solutions ($\bar{C}$).

In previous papers [24,31], we showed descriptions of the membrane transport of binary solutions of nonelectrolytes under conditions of heterogeneity of solutions by introducing the $R^{r}$ forms of the KKP equations and the R versions of the KKP equations for binary solutions of nonelectrolytes for the conditions of solution homogeneity. Here, we evaluated the transport properties of a membrane for aqueous ethanol solutions and the conditions of CP using network KKP equations. We experimentally determined the time and concentration characteristics of the volume ($J_{v}^{r}$) and solute ($J_{s}^{r}$) fluxes for conditions of homogeneity and CP. Next, we calculated the time and concentration dependencies of the CP coefficients ($\zeta_{v}^{r}$ and $\zeta_{s}^{r}$) and resistance coefficients ($R_{ij}^{r}$ and $R_{det}^{r};$ *i*, *j* ∈ {1, 2}, *r* = A, B). We used $J_{v}^{r}$, $J_{s}^{r},$ and $R_{ij}^{r}$ to calculate the energy conversion efficiency coefficients (($e_{ij}^{r}{)}_{R}$) and the flux of dissipated energy (*S*-energy) (($\Phi_{S}^{r}{)}_{R}$). Then, we used ($e_{ij}^{r}{)}_{R}$ and ($\Phi_{S}^{r}{)}_{R}$ to calculate the flux of free energy (*F*-energy) (($\Phi_{F}^{r}{)}_{R}$) and the flux of internal energy (*U*-energy) (($\Phi_{U}^{r}{)}_{R}$).

## 2. Materials and Methods

### 2.1. Membrane System

**Figure 1:**
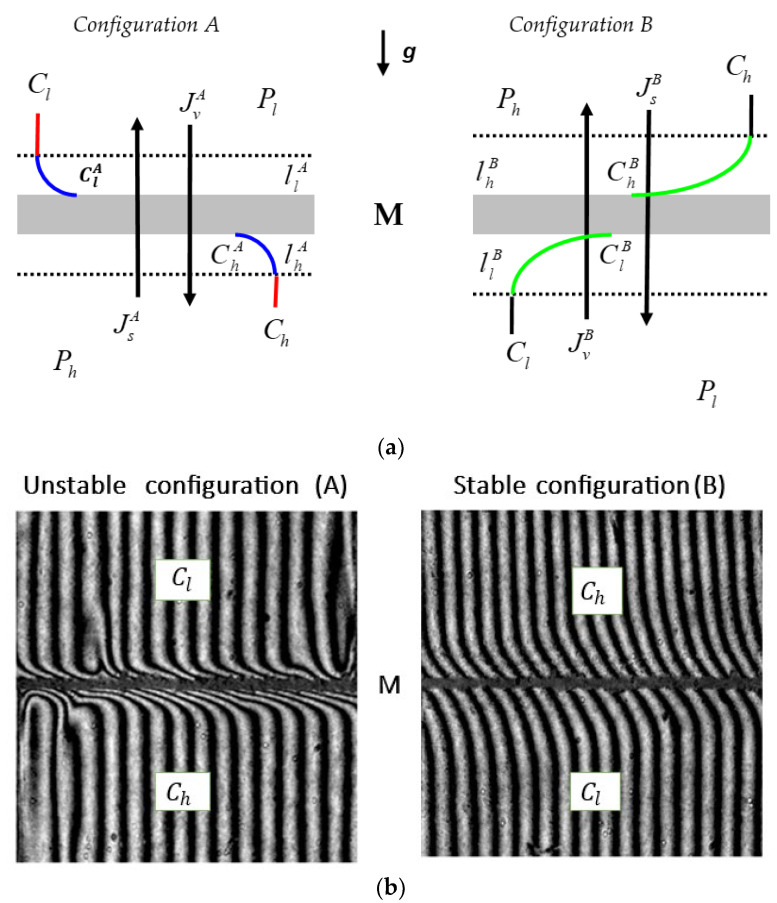
(**a**) The model of a single-membrane system: M—membrane; *g*—gravitational acceleration; $l_{l}^{A}$ and $l_{h}^{A}$ —the concentration boundary layers (CBLs) in configuration A; $l_{l}^{B}$ and $l_{h}^{B}$ —the CBLs in configuration B; *P_h_* and *P_l_*—mechanical pressures; $C_{h}$ and $C_{l}$ —total solution concentrations ($C_{h}$ > $C_{l}$ ); $C_{l}^{A}$, $C_{h}^{A}$, $C_{l}^{B}$, and $C_{h}^{B}$ —local (at boundaries between the membrane and CBLs) solution concentrations; $J_{v}^{A}$ —solute and volume fluxes in configuration A; $J_{v}^{B}$ —solute and volume fluxes in configuration B. (**b**) Interferometric images of concentration boundary layers for a membrane system that contains ethanol solutions of concentrations $C_{l}$ = 1 mol⋅m^−3^ and $C_{h}$ = 125 mol⋅m^−3^ at time 80 s; M—membrane [14].

The system used as a model to study membrane transport, illustrated schematically in Figure 1, consisted of a membrane (M) situated in the horizontal plane and separating two aqueous solutions of ethanol with concentrations at the initial moment of $C_{h}$ and $C_{l}$ = constant ($C_{h}$ ≥ $C_{l}$). The density of the solutions with concentrations of $C_{h}$ and $C_{l}$ fulfilled the condition $\rho_{h}$ ≤ $\rho_{l}$ = constant. In configuration A, a solution with the concentration $C_{l}$ was located in the compartment above the membrane and a solution with the concentration $C_{h}$ was in the compartment under the membrane. In configuration B, the solutions with the concentrations of $C_{l}$ and $C_{h}$ were swapped.

**Figure 2:**
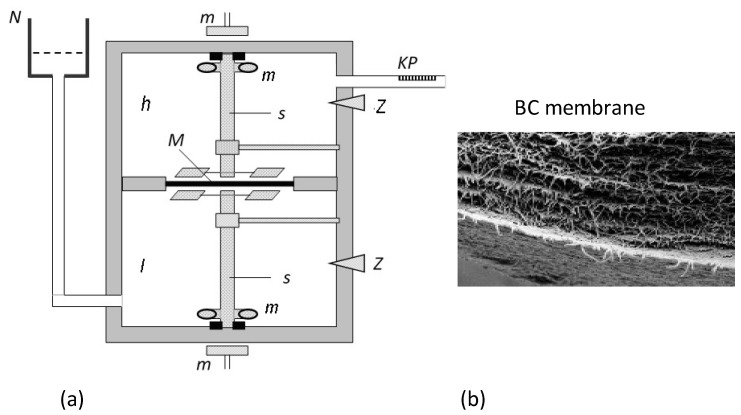
(**a**) Measuring system (*h* and *l*—measuring vessels, *N*—external solution tank, *s*—mechanical stirrers, *M*—membrane, *K*—calibrated pipette, *m*—magnets, Z—plugs) [33]. (**b**) Image of a cross section of a Bioprocess membrane obtained from a scanning electron microscope (magnification: 10,000 times) [37].

The study on volume ($J_{v}^{r}$) and solute ($J_{s}^{r}$) fluxes was carried out using the measuring set described in the paper [32] and is presented in Figure 2. It consisted of two cylindrical measuring vessels with volumes of 200 cm^3^ each containing aqueous ethanol solutions, one with a concentration in the range of 1–501 mol m^−3^ and the other with a constant concentration of 1 mol⋅m^−3^. The solutions in the vessels were separated by a previously described bacterial cellulose (BC) membrane called Bioprocess^®^ (Biofill Produtos Biotechnologicos S.A., Curitiba, Brasile) [33,34,35,36] positioned in a horizontal plane with an area of *A* = 3.36 cm^2^. The BC membrane was produced in flat sheets, and its structure was made of microcellulose fibers produced by *Acetobacter Xylinum* [8,37].

The volume flux was calculated based on the volume changes ($\Delta V^{r}$) in the pipette over time (Δ*t)* through the membrane surface (*A)* using the formula $J_{v}^{r}$ = ($\Delta V^{r}$)A^−1^(Δt)^−1^. The solute flux was calculated based on the formula $J_{s}^{r}$ = $\left(dC_{s}^{r}V_{u}\right)A$^−1^(Δt)^−1^, where $V_{u}$ is the volume of the measuring vessel and $dC_{s}^{r}$ is the increase in the total concentration of the solutions. The $dC_{s}^{r}$ was measured by a Rayleigh interferometer based on previously calculated feature curves, i.e., the experimental dependence of the shift of the interference bars (Δ*n*) as a function of the ethanol concentration (*C*) [38]. The study was carried out at T = 295 K. A laser interferometry method can also be used to determine $dC_{s}^{r}$ [39,40,41].

We measured $\Delta V^{r}$ and $dC_{s}^{r}$ under intense mechanical stirring of the solutions at 500 rpm, and when steady-state flows were obtained, the stirring of the solutions was turned off. In the second step, the increments of $\Delta V^{r}$ and $dC_{s}^{r}$ were measured until steady-state flows were obtained. The volume flux was from a vessel with a lower concentration of solutions to a vessel with a higher concentration of solutions, and the solute flux was in the opposite direction. The $\Delta V^{r}$ and $dC_{s}^{r}$ were measured in a series of independent experiments. From the measurements of $\Delta V^{r}$ and $dC_{s}^{r}$, the characteristics $J_{v}^{r}$ = *f*(*t*) and $J_{s}^{r}$ = *f*(*t*) were determined for different concentrations of ethanol solutions. For each characteristic, three independent experiments were performed. The relative error in the determination of $J_{v}^{r}$ = *f*(*t*) and $J_{s}^{r}$ = *f*(*t*) was no greater than 10%.

Based on the time characteristics of $J_{v}^{r}$ and $J_{s}^{r}$ for the steady state, we calculated the concentration characteristics of $J_{v}^{r}$ and $J_{s}^{r}$. Next, we used the characteristics $J_{v}$ = *f*(*t*) (for the homogeneity of solutions) and $J_{v}^{r}$ = *f*(*t*) (for the conditions of CP) to calculate the dependence $\zeta_{v}^{r}=f(t$). Similarly, we used the characteristics of $J_{s}$ = *f*(*t*) (for the homogeneity of solutions) and $J_{s}^{r}$ = (t) (for CP conditions) to calculate the dependence $\zeta_{s}^{r}$ = *f*(*t*). Additionally, the dependence $\zeta_{v}^{r}=f(\Delta C$) was determined based on the characteristics $J_{v}$ = *f*(Δ*C*) (for the homogeneity conditions of solutions and $J_{v}^{r}$ = *f*(ΔC) for CP conditions. Similarly, the dependence $\zeta_{s}^{r}=f(\Delta C$) was determined based on the characteristics $J_{s}$ = *f*(ΔC) for the conditions of CP and $J_{s}^{r}$ = *f*(ΔC) for CP conditions. Moreover, the dependences $R_{ij}^{r}=f(\Delta C$), $R_{det}^{r}=f(\Delta C$), ${\left(e_{ij}^{r}\right)}_{R}=f(\Delta C$), ${\left(\phi_{ij}^{r}\right)}_{R}=f(\Delta C$), ${\left(\varphi_{ij}\right)}_{R}=f(\Delta C$), and ${\left(\Phi_{S}^{r}\right)}_{R}=f(\Delta C$) were calculated.

### 2.2. The R^r^ Form of Kedem–Katchalsky Equations for Binary Nonelectrolyte Solutions

For the interpretation of the obtained results, we used the *R^r^* form of the KKP equations, which can be obtained using simple algebraic transformations presented in the paper [24,31]:

(3) $$∆P-∆\pi =\left(\frac{\bar{C}\left(1-\zeta_{v}^{r}\sigma_{v}\right)\left(1-\zeta_{a}^{r}\sigma_{s}\right)}{\zeta_{p}^{r}L_{p}\zeta_{s}^{r}\omega }\right)J_{v}^{r}-\frac{1}{\zeta_{s}^{r}\omega }\left(1-\zeta_{v}^{r}\sigma_{v}\right)J_{s}^{r}$$

(4) $$\frac{∆\pi }{\bar{C}}=-\frac{1}{\zeta_{s}^{r}\omega }\left(1-\zeta_{a}^{r}\sigma_{s}\right)J_{v}^{r}+\frac{1}{\bar{C}\zeta_{s}^{r}\omega }J_{s}^{r}.\;$$

The above equations can be written in matrix form:

(5) $$\left[\begin{array}{c} ∆P-∆\pi \\ \frac{∆\pi }{\bar{C}} \end{array}\right]=\left[R^{r}\right]\left[\begin{array}{c} J_{v}^{r}\\ J_{s}^{r} \end{array}\right]$$

wre $\left[R^{r}\right]$ is the matrix of resistance coefficients given by

(6) $$\left[R^{r}\right]=\left[\begin{array}{cc} R_{11}^{r} & R_{12}^{r}\\ R_{21}^{r} & R_{22}^{r} \end{array}\right]=\left[\begin{array}{cc} \frac{\zeta_{s}^{r}\omega +\zeta_{p}^{r}L_{p}∆C\left(1-\zeta_{v}^{r}\sigma_{v}\right)\left(1-\zeta_{a}^{r}\sigma_{s}\right)ln(C_{h}C_{l}{}^{-1})}{\zeta_{p}^{r}L_{p}\zeta_{s}^{r}\omega } & -\frac{1}{\zeta_{s}^{r}\omega }\left(1-\zeta_{v}^{r}\sigma_{v}\right)\\ -\frac{1}{\zeta_{s}^{r}\omega }\left(1-\zeta_{a}^{r}\sigma_{s}\right) & \frac{ln(C_{h}C_{l}{}^{-1})}{\zeta_{s}^{r}\omega ∆C} \end{array}\right]$$

From the above equation, it follows that $R_{12}^{r}$ ≠ $R_{21}^{r}$ and that the matrix determinant $\left[R^{r}\right]$ is equal to

(7) $$R_{det}^{r}=det\left[R^{r}\right]=\frac{ln(C_{h}C_{l}{}^{-1})}{\zeta_{p}^{r}L_{p}\zeta_{s}^{r}\omega ∆C}$$

To write Equations (3)–(7) for the homogeneity conditions of the solutions, it is enough to leave the ‘*r*’ index and assume $\zeta_{p}^{r}$ = $\zeta_{v}^{r}$ = $\zeta_{s}^{r}$ = $\zeta_{a}^{r}$ = 1. Then, we have $R_{ij}^{r}$ = $R_{ij}$ and $R_{det}^{r}$ = $R_{det}$.

The first part of the right-hand side of Equation (4) has the sense of the membrane Peclét number [42]. The classical definition of this number has the form $Pe=\left(1-\sigma \right)J_{v}{℘}^{-1}$ = $R_{21}J_{v}$ and appears in Equation (4) for conditions of homogeneity of solutions ($\zeta_{s}^{r}$ = $\zeta_{a}^{r}$ = 1, $J_{v}^{r}$ = $J_{v}$, and $\sigma_{s}$ = σ). In this equation $\left(1-\sigma \right){℘}^{-1}$ ≡ α is the Peclét coefficient and ℘ is the solute permeability coefficient. For conditions of concentration polarization, this number can be written in the form

(8) $${\left(Pe\right)}_{v}^{r}=\frac{\left(1-\zeta_{a}^{r}\sigma_{s}\right)J_{v}^{r}}{\zeta_{s}^{r}\omega RT}=\alpha_{v}^{r}J_{v}^{r}=\frac{R_{21}^{r}}{RT}J_{v}^{r}$$

where $\omega RT={℘}_{v}$ and $\alpha_{v}^{r}$ is expressed in s m^−1^.

The second part of the right side of Equation (3) is similar to the membrane Peclét number. However, in this case, the definition of this number has the form $Pe=\left(1-\sigma \right)J_{s}{℘}^{-1}$ = $R_{12}J_{s}$ and appears in Equation (3) for conditions of homogeneity of solutions ($\zeta_{s}^{r}$ = $\zeta_{v}^{r}$ = 1, $J_{s}^{r}$ = $J_{s}$, and $\sigma_{v}$ = σ). For conditions of concentration polarization, this number can be written in the form

(9) $${\left(Pe\right)}_{s}^{r}=\frac{\left(1-\zeta_{v}^{r}\sigma_{v}\right)J_{s}^{r}}{\zeta_{s}^{r}\omega RT\bar{C}}=\alpha_{s}^{r}J_{s}^{r}=\frac{R_{12}^{r}}{RT\bar{C}}J_{s}^{r}$$

where $\omega RT\bar{C}$ = ${℘}_{s}$ and $\alpha_{s}^{r}$ is expressed in m^2^s mol^−1^.

It follows that the coefficients $R_{21}$ = $a_{v}RT$, $R_{12}$ = $a_{s}RT\bar{C}$, $R_{21}^{r}$ = $\alpha_{v}^{r}RT,$ and $R_{12}^{r}$ = $\alpha_{s}^{r}RT\bar{C}$ (*r* = A, B) are related to Peclét’s coefficients, which are known from the literature [24,42].

Using the coefficients $R_{ij}^{r}$, $R_{ij}$, $R_{det}^{r},$ and $R_{det}$, it is possible to define the coefficients ${\left(\phi_{ij}^{r}\right)}_{R}$ and ${\left(\phi_{det}^{r}\right)}_{R}$, which are measures of the CP effect, and the coefficients ${\left(\varphi_{ij}\right)}_{R}$ and ${\left(\varphi_{det}\right)}_{R}$, which are measures of the effect of gravitational convection in osmotic and diffusive membrane transport. The definitions of these coefficients can be written as

(10) $${\left(\phi_{ij}^{r}\right)}_{R}=\frac{R_{ij}^{r}}{R_{ij}}$$

(11) $${\left(\phi_{det}^{r}\right)}_{R}=\frac{R_{det}^{r}}{R_{det}}$$

(12) $${\left(\varphi_{ij}\right)}_{R}=\frac{R_{ij}^{A}-R_{ij}^{B}}{R_{ij}}$$

(13) $${\left(\varphi_{det}\right)}_{R}=\frac{R_{det}^{A}-R_{det}^{B}}{R_{det}}$$

The coefficients $\phi_{ij}^{r}$ and $\phi_{det}^{r}$ are measures of the distance of the membrane system from the CP state, and the coefficients $\varphi_{ij}$ and $\varphi_{det}$ are measures of the distance of the membrane system from the unstable state.

In thermodynamic systems, including membrane systems, *U*-energy can be converted into *F*-energy and *S*-energy (*TS*) [3,22]. If the solutions contain a solvent and one solute, the flux of *S*-energy for the CP conditions ($\Phi_{S}^{r}$) is described by the equation [5]

(14) $${\left(\Phi_{S}^{r}\right)}_{R}={\left[{\left(\Phi_{S}^{r}\right)}_{R}\right]}_{J_{v}^{r}}+{\left[{\left(\Phi_{S}^{r}\right)}_{R}\right]}_{J_{s}^{r}}=J_{v}^{r}\left(∆P-∆\pi \right)+J_{s}^{r}\frac{∆\pi }{\bar{C}}$$

where ${\left(\Phi_{S}^{r}\right)}_{R}$ is the global *S*-energy for CP conditions, ${\left[{\left(\Phi_{S}^{r}\right)}_{R}\right]}_{J_{v}^{r}}$ is the *S*-energy produced by $J_{v}^{r}$, and ${\left[{\left(\Phi_{S}^{r}\right)}_{R}\right]}_{J_{s}^{r}}$ is the *S*-energy produced by $J_{s}^{r}$.

Taking into account Equations (3) and (4), in Equation (14) we obtain

(15) $${\left(\Phi_{S}^{r}\right)}_{R}=\left[R_{11}^{r}{\left(J_{v}^{r}\right)}^{2}+\left(R_{12}^{r}+R_{21}^{r}\right)J_{v}^{r}J_{s}^{r}+R_{22}^{r}{\left(J_{s}^{r}\right)}^{2}\right]$$

An explicit form of the coefficients $R_{11}^{r}$, $R_{12}^{r}$, $R_{21}^{r}$, and $R_{22}^{r}$ appearing in the above equation is given in Equation (6). To obtain a global *S*-energy for the conditions of homogeneity of solutions ($\Phi_{S}$) in Equations (14) and (15), one should assume the condition of $\zeta_{p}^{r}$ = $\zeta_{s}^{r}$ = $\zeta_{v}^{r}$ = $\zeta_{a}^{r}$ = 1. In turn, from Equation (6), the coefficients ${\left(e_{ij}^{r}\right)}_{R}$, $r_{ij}^{r},$ and $Q_{R}^{r}$ can be expressed using the coefficients $R_{ij}^{r}$.

Using the definition proposed by Kedem and Caplan [43] and Peusner [30], we present the definitions of the energy conversion efficiency coefficients for CP conditions:

(16) $${\left(e_{ij}^{r}\right)}_{R}=\frac{{\left(R_{ij}^{r}\right)}^{2}}{R_{ii}^{r}R_{jj}^{r}{\left(1+\sqrt{1-\frac{R_{ij}^{r}R_{ji}^{r}}{R_{ii}^{r}R_{jj}^{r}}}\right)}^{2}}=\frac{{\left(r_{ij}^{r}\right)}^{2}}{{\left(1+\sqrt{1-r_{ij}^{r}r_{ji}^{r}}\right)}^{2}}$$

where $r_{ij}^{r}=-R_{ij}^{r}/{\left(R_{ii}^{r}R_{jj}^{r}\right)}^{0.5}$ is the coupling coefficient [30,43]. To obtain the expressions for $r_{ij}$ and $r_{ji}$, it is enough to omit the superscripts “*r*” due to the fact that $R_{ij}^{r}$ ≈ $R_{ji}^{r}$, $R_{ij}$ = $R_{ji}$, $r_{ij}^{r}$ ≠ $r_{ji}^{r}$, and $r_{ij}$ = $r_{ji}$. In turn, to obtain the expressions for ${\left(e_{ij}\right)}_{R}$ and ${\left(e_{ji}\right)}_{R}$, it is enough to omit the superscripts “*r*” due to the fact that $r_{ij}^{r}$ ≈ $r_{ji}^{r},$ $r_{ij}$ = $r_{ji}$, ${\left(e_{ij}^{r}\right)}_{R}$ ≈ ${\left(e_{ji}^{r}\right)}_{R}$, and ${\left(e_{ij}\right)}_{R}$ = ${\left(e_{ji}\right)}_{R}$.

According to the first law of thermodynamics, for isothermal isochoric processes, the following equation is correct:

(17) $${\left(\Phi_{U}^{r}\right)}_{R}={\left(\Phi_{F}^{r}\right)}_{R}+{\left(\Phi_{S}^{r}\right)}_{R}$$

where$\;{\left(\Phi_{S}^{r}\right)}_{R}=A^{-1}Td_{i}S^{r}/dt$ is the flux of dissipated energy (*S*-energy), ${\left(\Phi_{F}^{r}\right)}_{R}=A^{-1}dF^{r}/dt$ is the flux of free energy (*F*-energy), and ${\left(\Phi_{U}^{r}\right)}_{R}=A^{-1}dU^{r}/dt$ is the flux of internal energy (*U*-energy). All of these fluxes are expressed in Wm^−2^.

We calculate the fluxes ${\left(\Phi_{F}^{r}\right)}_{R}$ and ${\left(\Phi_{S}^{r}\right)}_{R}$ using the expression below:

(18) $${\left(e_{max}{}^{r}\right)}_{R}=\frac{{\left(\Phi_{F}^{r}\right)}_{R}}{{\left(\Phi_{F}^{r}\right)}_{R}+{\left(\Phi_{S}^{r}\right)}_{R}}=1-\frac{{\left(\Phi_{F}^{r}\right)}_{R}}{{\left(\Phi_{S}^{r}\right)}_{R}}$$

Transforming this expression, we obtain

(19) $${\left(\Phi_{F}^{r}\right)}_{R}=\frac{{\left(e_{max}\right)}_{R}}{1-{\left(e_{max}\right)}_{R}}{\left(\Phi_{S}^{r}\right)}_{R}$$

(20) $${\left(\Phi_{U}^{r}\right)}_{R}=\frac{1}{1-{\left(e_{max}\right)}_{R}}{\left(\Phi_{S}^{r}\right)}_{R}$$

The transport properties of the BC membrane were determined by the hydraulic permeability ($L_{p}$), reflection (σ), and solute permeability (ω) coefficients. The values of these coefficients, determined in a series of independent experiments that were carried out according to a previously described procedure [14], were $L_{p}$ = (62.8 ± 0.5) × 10^−12^ m^3^ N^−1^s^−1^, σ = (0.23 ± 0.01) × 10^−2^, and ω = (15.3 ± 0.5) × 10^−10^ mol N^−1^s^−1^.

## 3. Results and Discussion

### 3.1. The Time and Concentration Dependencies of $J_{v}^{r}$ and $J_{s}^{r}$

**Figure 3:**
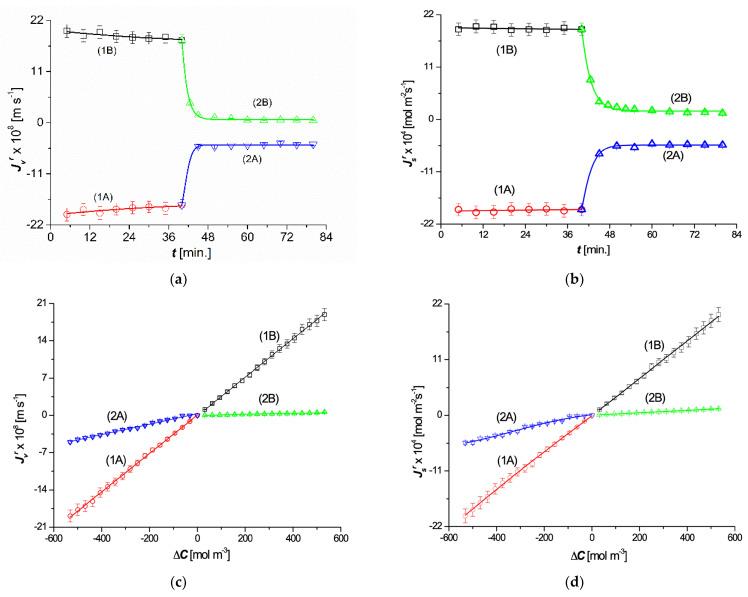
Dependences $J_{v}^{r}=f\left(t\right)\;$ (**a**), $J_{s}^{r}=f\left(t\right)$ (**b**), $J_{v}^{r}=f\left(∆C\right)$ (**c**)**,** and $J_{s}^{r}=f\left(∆C\right)$ (**d**): curves 1A and 1B were obtained for homogeneous solutions (mechanical mixing), and curves 2A and 2B were obtained for concentration polarization conditions (after excluding mechanical mixing of the solutions).

The time dependencies of the volume flux ($J_{v}^{r}$) and the solute flux ($J_{s}^{r}$) for $C_{h}$ = 501 mol m^−3^ and $C_{l}$ = 1 mol m^−3^ are shown in Figure 3a,b. Curves 1A and 1B were obtained for mechanically stirred solutions that favored solution homogeneity. Curves 1A and 1B are symmetrical with respect to the horizontal axes passing through the points $J_{v}^{r}$ = 0 and $J_{s}^{r}$ = 0, indicating that stirring was effective. This symmetry is reflected in the linearity of the dependences $J_{v}^{r}=f\left(∆C\right)$ and $J_{s}^{r}=f\left(∆C\right)$, as illustrated by curves 1A and 1B in Figure 3c,d. In steady states, the relations $\left|J_{v}^{A}\right|$ = $J_{v}^{B}$ = $J_{v}$ and $\left|J_{s}^{A}\right|$ = $J_{s}^{B}$ = $J_{s}$ were fulfilled. In CP conditions, the time dependencies of $J_{v}^{r}$ and $J_{s}^{r},$ shown by curves 2A and 2B, are asymmetric with respect to the horizontal axes passing through the points $J_{v}^{r}$ = 0 and $J_{s}^{r}$ = 0. The consequence of this asymmetry is the nonlinear dependencies $J_{v}^{r}=f\left(∆C\right)$ and $J_{s}^{r}=f\left(∆C\right),$ illustrated by curves 2A and 2B in Figure 3c,d. The shapes of these graphs indicate that both $J_{v}^{r}$ and $J_{s}^{r}$ reached steady states relatively quickly and that in the steady states $\left|J_{v}^{A}\right|$ > $J_{v}^{B}$ and $\left|J_{s}^{A}\right|$ > $J_{s}^{B}$. This dependence was a consequence of the emergence of gravitational convection, which is destructive to CBLs. This means that, in this case, CP and gravitational convection were antagonistic processes.

The characteristics of $J_{v}^{B}=f\left(t\right)$, $J_{s}^{B}=f\left(t\right)$, $J_{v}^{B}=f\left(∆C\right),$ and $J_{s}^{B}=f\left(∆C\right)$ presented in Figure 3a–d, illustrated by plots 2B, are typical for solutions whose densities decrease with increasing concentrations and CP conditions. Examples include aqueous solutions of ethanol, methanol, or ammonia [13]. If an aqueous solution of such a substance is placed in the compartment above the membrane (configuration B), a stable system of CBLs is formed, which causes a reduction in the value of the osmotic pressure difference, which results in the $J_{v}^{B}$ and $J_{s}^{B}$ fluxes. In configuration A, which refers to the situation when an aqueous solution of such a substance is placed in a compartment under the membrane, natural convection occurs, which decreases the reduction in the value of the osmotic pressure difference and causes fluxes $J_{v}^{A}$ and $J_{s}^{A}$.

### 3.2. The Time and Concentration Dependencies of $\zeta_{v}^{r}$ and $\zeta_{s}^{r}$

**Figure 4:**
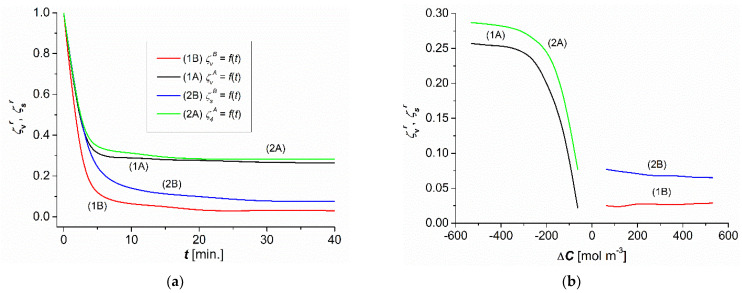
Time (**a**) and concentration (**b**) dependences of $\zeta_{v}^{r}$ and $\zeta_{s}^{r}$ for aqueous ethanol solutions.

The time dependencies of $\zeta_{v}^{r}$ and $\zeta_{s}^{r}$ were calculated based on the results illustrated in Figure 4a,b. According to the definitions of $\zeta_{v}^{r}$ = $J_{v}^{r}$/$J_{v}$ and $\zeta_{s}^{r}$ = $J_{s}^{r}$/$J_{s}$, to obtain the dependencies presented in Figure 4a, ${\left[\zeta_{v}^{r}\left(t\right)\right]}_{∆C=const.}$ = ${\left[J_{v}^{r}\left(t\right)/J_{v}\left(t\right)\right]}_{∆C=const.}$ and ${\left[\zeta_{s}^{r}\left(t\right)\right]}_{∆C=const.}$ = ${\left[J_{s}^{r}\left(t\right)/J_{s}\left(t\right)\right]}_{∆C=const.}$ for ∆C = 500 mol m^−3^. The coefficients $\zeta_{v}^{r}$ and $\zeta_{s}^{r}$ take their values from the intervals ${\left(\zeta_{v}^{r}\right)}_{diff.}$ ≤ $\zeta_{v}^{r}$ ≤ 1, ${\left(\zeta_{v}^{r}\right)}_{conv.}$ ≤ $\zeta_{v}^{r}$ ≤ 1, ${\left(\zeta_{s}^{r}\right)}_{diff.}$ ≤ $\zeta_{s}^{r}$ ≤ 1, and ${\left(\zeta_{s}^{r}\right)}_{conv.}$ ≤ $\zeta_{s}^{r}$ ≤ 1. As shown in Figure 4a, $\zeta_{v}^{r}$ and $\zeta_{s}^{r}$ take values from the intervals 0.03 ≤ $\zeta_{v}^{r}$ ≤ 1, 0.26 ≤ $\zeta_{v}^{r}$ ≤ 1, 0.06 ≤ $\zeta_{s}^{r}$ ≤ 1, and 0.29 ≤ $\zeta_{s}^{r}$ ≤ 1. Based on these time dependencies, the concentration dependencies $\zeta_{v}^{r}$ = *f*(∆C) and $\zeta_{s}^{r}$ = *f*(∆C) were determined for the steady states (Figure 4b). The coefficients $\zeta_{v}^{r}$ and $\zeta_{s}^{r}$ take their values from the intervals ${\left(\zeta_{v}^{r}\right)}_{diff.}$ ≤ $\zeta_{v}^{r}$ ≤ ${\left(\zeta_{v}^{r}\right)}_{conv.}$ and ${\left(\zeta_{s}^{r}\right)}_{diff.}$ ≤ $\zeta_{s}^{r}$ ≤ ${\left(\zeta_{s}^{r}\right)}_{conv.}$. As shown in Figure 4b, $\zeta_{v}^{r}$ and $\zeta_{s}^{r}$ take their values in the range between 0.03 ≤ $\zeta_{v}^{r}$ ≤ 0.26 and 0.06 ≤ $\zeta_{s}^{r}$ ≤ 0.29. Therefore, the coefficients $\zeta_{v}^{r}$ and $\zeta_{s}^{r}$ are a measure of the CP in both convection and nonconvection states.

The transition from nonconvective to convective states is controlled by the Rayleigh concentration number ($R_{C}$). The critical value of this number can be calculated from Equation (18):

(21) $$R_{C}=\frac{gD^{2}∆C}{16R^{3}T^{3}\omega^{3}\rho_{0}\nu_{0}}\left(\frac{\partial \rho }{\partial C}\right)\frac{{\left(1-\zeta \right)}^{4}}{\zeta^{3}}$$

where g is the gravitational acceleration, *D* is the diffusion coefficient in the solution, $\rho_{0}$ is the mass density, $\nu_{0}$ is the kinematic viscosity of the solution, ω is the solute permeability coefficient through the membrane, ∂ρ/∂C is the variation in density with concentration, and ζ is the concentration polarization coefficient.

Considering g = 9.81 m s^−2^, RT = 24.51 × 10^2^ J mol^−1^, $\rho_{0}$ = 998.2 kg m^−3^, $\nu_{0}$ = 1.01 × 10^−6^ m^2^s^−1^, ω = 1.53 × 10^−9^ mol N^−1^s^−1^, D = 1.07 × 10^−9^ m^2^s^−1^, ∂ρ/∂C = −0.009 kg mol^−1^, ∆C = 80 mol m^−3^, and ζ = 0.16 (estimated based Figure 4b) in Equation (19), we obtain $R_{C}$ = −1155.07. The minus sign indicates that the convective currents are directed vertically upwards. In contrast, for aqueous glucose solutions, studied previously, convective currents were directed vertically downwards, and therefore $R_{C}$ had a positive sign [5]. The obtained critical value of $R_{C}$ is consistent with the values presented in the papers [44,45].

### 3.3. Concentration Dependencies of the Resistance Coefficients $R_{ij}^{r}$ and $R_{det}^{r}$

**Figure 5:**
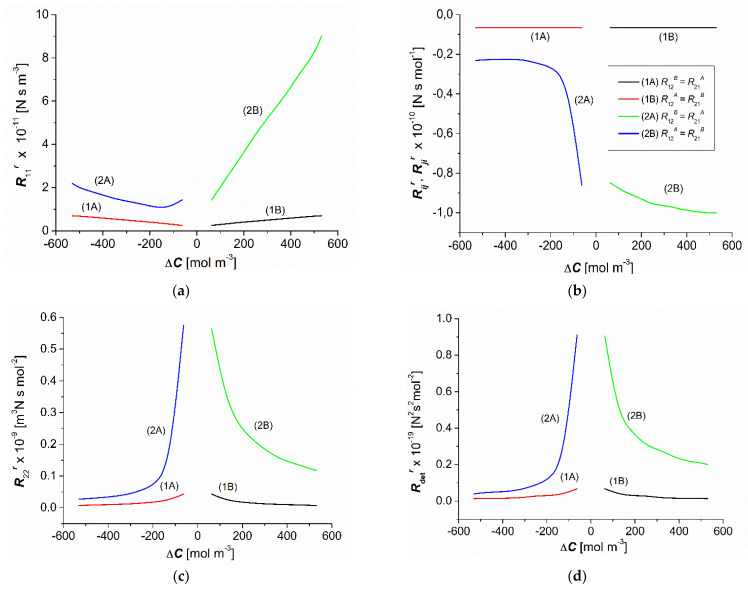
Concentration dependences of the resistance coefficients (**a**) $R_{11}^{r},$ (**b**) $R_{12}^{r}=R_{21}^{r}\;$, (**c**) $\;R_{22}^{r}$, and (**d**) $R_{det}^{A}$ for aqueous ethanol solutions.

The concentration dependencies of the resistance coefficients $R_{11}^{r}$*,* $R_{12}^{r}$*,* $R_{21}^{r}$*,* $R_{22}^{r},$ and $R_{det}^{r}$, calculated based on Equations (6) and (7), are shown in Figure 5a–d for homogenous solutions (curves 1A and 1B) and CP conditions (curves 2A and 2B). For all studied dependences of the resistance coefficients, curves 1A and 1B were symmetrical, whereas curves 2A and 2B were asymmetrical with respect to the point ΔC = 0.

The comparison of the dependencies $R_{11}^{r}$ = *f*(ΔC) (Figure 5a) for homogenous and CP conditions indicates that for $\left|-∆C\right|$ = ∆C the condition $R_{11}^{A}$ = $R_{11}^{B}$ = $R_{11}$ was fulfilled. In turn, for the same values (−∆C), the condition $R_{11}^{A}$ > $R_{11}$ was fulfilled, and for the same values of ∆C the condition $R_{11}^{B}$ > $R_{11}$. Moreover, for $\left|-∆C\right|$ = ∆C the condition $R_{11}^{A}$ < $R_{11}^{B}$ was fulfilled, and for $\left|-∆C\right|$ = ∆C = 62.5 mol m^−3^ the condition $R_{11}^{A}$ = $R_{11}^{B}$ was fulfilled.

The dependencies $R_{ij}^{r}$ = *f*(Δ*C*) and $R_{ji}^{r}$ = *f*(ΔC), presented in Figure 5b, indicate that for $\left|-∆C\right|$ = ∆C, the conditions were $R_{12}^{A}$ = $R_{21}^{A}$, $R_{12}^{B}$ = $R_{21}^{B},$ and $R_{12}$ = $R_{21}$. In turn, for the same values (−∆C), the condition $R_{12}^{A}$ = $R_{21}^{A}$ < $R_{12}$ = $R_{21}$ was fulfilled, and for the same values of ∆C the condition $R_{12}^{B}$ = $R_{21}^{B}$ < $R_{12}$ = $R_{21}$ was fulfilled. Moreover, for $\left|-∆C\right|$ = ∆C the condition was $R_{12}^{A}$ = $R_{21}^{A}$ > $R_{12}^{B}$ = $R_{21}^{B}$, and for $\left|-∆C\right|$ = ∆C = 62.5 mol m^−3^ the condition was $R_{12}^{A}$ = $R_{21}^{A}$ = $R_{12}^{B}$ = $R_{21}^{B}$. From Equation (6), it follows that $R_{12}^{r}$ ≠ $R_{21}^{r}$. To explain why this relation did not hold, we calculated the quotient $R_{12}^{r}$/$R_{21}^{r}$ = $\left(1-\zeta_{v}^{r}\sigma \right)/\left(1-\zeta_{a}^{r}\sigma \right)$ using Equation (5), and we obtained $R_{12}^{r}$/$R_{21}^{r}$ = 1.002, meaning that $R_{12}^{A}$ = $R_{21}^{A}$, with accuracy to two significant figures.

As shown in Figure 5c, the dependencies of $R_{22}^{r}$ = *f*(Δ*C*) indicate that for $\left|-∆C\right|$ = ∆C the condition $R_{22}^{A}$ = $R_{22}^{B}$ = $R_{22}$ was fulfilled. In turn, for the same values (−∆C), the condition $R_{22}^{A}$ > $R_{22}$ was fulfilled, and for the same values of ∆C the condition $R_{22}^{B}$ > $R_{22}$ was fulfilled. Moreover, for $\left|-∆C\right|$ = ∆C the condition $R_{22}^{A}$ < $R_{22}^{B}$ was fulfilled, and for $\left|-∆C\right|$ = ∆C = 62.5 mol m^−3^ the condition $R_{22}^{A}$ = $R_{22}^{B}$ was fulfilled.

The dependencies of $R_{det}^{r}$ = *f*(Δ*C*), shown in Figure 5d, indicate that for $\left|-∆C\right|$ = ∆C the condition $R_{det}^{A}$ = $R_{det}^{B}$ = $R_{det}$ was satisfied. In turn, for the same values (−∆C), the condition $R_{det}^{A}$ > $R_{det}$ was fulfilled, and for the same values of ∆C—the condition $R_{det}^{B}$ > $R_{det}$. Moreover, for $\left|-∆C\right|$ = ∆C the condition $R_{det}^{A}$ < $R_{det}^{B}$ was fulfilled, and for $\left|-∆C\right|$ = ∆C = 62.5 mol m^−3^ the condition $R_{det}^{A}$ = $R_{det}^{B}$ was fulfilled.

The coefficients $R_{11}^{r}$, $R_{22}^{r},$ and $R_{det}^{r}$ were positive, and the coefficients $R_{12}^{r}$ and $R_{21}^{r}$ were negative and dependent on ∆C for the CP conditions. Compared to the conditions of homogeneity of solutions, the CP increased the value of the coefficients $R_{11}^{r}$, $R_{22}^{r}$, and $R_{det}^{r}$ and reduced the value of the coefficients $R_{12}^{r}$ and $R_{21}^{r}$. For the same ∆C, the values of the coefficients $R_{11}^{r}$, $R_{22}^{r}$, and $R_{det}^{r}$ were smaller, and the coefficients $R_{12}^{r}$ and $R_{21}^{r}$ were higher for the nonconvective state.

Using the dependencies shown in Figure 3c,d and Figure 5a–c, we calculated the values of the Peclét numbers ${\left(Pe\right)}_{v}$, ${\left(Pe\right)}_{v}^{r}$, ${\left(Pe\right)}_{s},$ and ${\left(Pe\right)}_{s}^{r}$ for the point at which $J_{v}$ = 2.83 × 10^−8^ m s^−1^, $J_{v}^{A}$ = $J_{v}^{B}$ = 0.45 × 10^−8^ m s^−1^, $J_{s}$ = 3.01 × 10^−4^ mol m^−2^s^−1^, and $J_{s}^{A}$ = $J_{s}^{B}$ = 0.48 × 10^−4^ mol m^−2^s^−1^. The values of these Peclét numbers were ${\left(Pe\right)}_{v}$ = 7.5 ×10^−6^, ${\left(Pe\right)}_{s}$ = 7.5 × 10^−3^, ${\left(Pe\right)}_{v}^{r}$ = 7.99 × 10^−2^, and ${\left(Pe\right)}_{s}^{r}$ = 79.95, and ${\left(Pe\right)}_{s}^{r}$ > ${\left(Pe\right)}_{v}^{r}$ > ${\left(Pe\right)}_{s}$ > ${\left(Pe\right)}_{v}$.

### 3.4. Concentration Dependencies ${\left(\phi_{ij}^{r}\right)}_{R}$ and ${\left(\phi_{det}^{r}\right)}_{R}$

**Figure 6:**
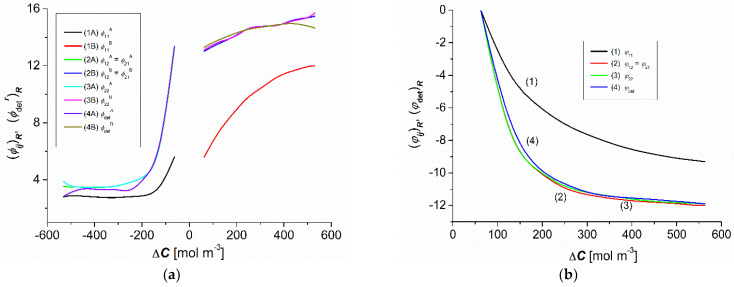
Concentration dependencies of the coefficients ${\left(\phi_{ij}^{r}\right)}_{R}$ and ($\phi_{det}^{r}{)}_{R}$ (**a**) and the coefficients ${\left(\varphi_{ij}\right)}_{R}$ and ($\varphi_{det}{)}_{R}$ (**b**) for aqueous ethanol solutions.

Figure 6a,b shows the concentration dependencies of the CP effects ${\left(\phi_{ij}^{r}\right)}_{R}$ and ${\left(\phi_{det}^{r}\right)}_{R}$, calculated based on Equations (10) and (11). Figure 6a shows that for the same indices *ij* the dependencies ${\left(\phi_{ij}^{r}\right)}_{R}$ = *f*(–∆C) were asymmetric to the dependence ${\left(\phi_{ij}^{r}\right)}_{R}$ = *f*(∆C). The plots 2A, 3A, and 4A overlap for negative ∆C values, whereas plots 2B, 3B, and 4B overlap for positive ∆C values. This means that ${\left(\phi_{12}^{A}\right)}_{R}$ ≈ ${\left(\phi_{21}^{A}\right)}_{R}$≈ ${\left(\phi_{22}^{r}\right)}_{R}$ ≈ ${\left(\phi_{det}^{A}\right)}_{R}$ > ${\left(\phi_{11}^{A}\right)}_{R}$ and ${\left(\phi_{12}^{B}\right)}_{R}$ ≈ ${\left(\phi_{21}^{B}\right)}_{R}$ ≈ ${\left(\phi_{22}^{B}\right)}_{R}$ ≈ ${\left(\phi_{det}^{B}\right)}_{R}$ > ${\left(\phi_{11}^{B}\right)}_{R}$. Figure 6b shows the concentration dependencies of the effect of gravitational convection, ${\left(\varphi_{ij}\right)}_{R}$ = *f*(∆C) and ${\left(\varphi_{det}\right)}_{R}$ = *f*(∆C), calculated based on Equations (12) and (13).

As shown in Figure 6b, for the same ∆C values of the dependency, ${\left(\varphi_{12}\right)}_{R}$ ≈ ${\left(\varphi_{21}\right)}_{R}$ ≈ ${\left(\varphi_{22}\right)}_{R}$ ≈ ${\left(\varphi_{det}\right)}_{R}$ < ${\left(\varphi_{11}\right)}_{R}$. Moreover, the values of the coefficients ${\left(\varphi_{12}\right)}_{R}$, ${\left(\varphi_{21}\right)}_{R},$ ${\left(\varphi_{22}\right)}_{R}$, ${\left(\varphi_{det}\right)}_{R}$, and ${\left(\varphi_{11}\right)}_{R}$ were negative. According to the convention adopted in [5], the convection currents in a system containing aqueous ethanol solutions, which are a consequence of the hydrodynamic instabilities of CBLs, are directed vertically upwards. In contrast, the convection currents in a system containing aqueous glucose solutions are directed vertically downward. The results from Equations (12) and (13) state that for the nonconvective state ${\left(\varphi_{ij}\right)}_{R}$ = 0 and ($\varphi_{det}{)}_{R}$ = 0 and for the convective state ${\left(\varphi_{ij}\right)}_{R}$ < 0 and ($\varphi_{det}{)}_{R}$ < 0. Therefore, from Figure 6b we can conclude that ${\left(\varphi_{ij}\right)}_{R}$ = 0 and ($\varphi_{det}{)}_{R}$ = 0 for ΔC = 80 mol m^−3^.

The coefficients ${\left(\phi_{ij}^{r}\right)}_{R}$ and ($\phi_{det}^{r}{)}_{R}$ were positive, and the coefficients ${\left(\varphi_{ij}\right)}_{R}$ and ($\varphi_{det}{)}_{R}$ were negative. The negative convection effect shows that the convection movements were directed vertically upwards. Moreover, for the same ∆C, the values of these coefficients were higher for the nonconvective state.

### 3.5. Concentration Dependencies of ${\left(\Phi_{S}^{r}\right)}_{R}$, ${\left(e_{ij}^{r}\right)}_{R}$, ${\left(\Phi_{F}^{r}\right)}_{R}$, and ${\left(\Phi_{U}^{r}\right)}_{R}$

**Figure 7:**
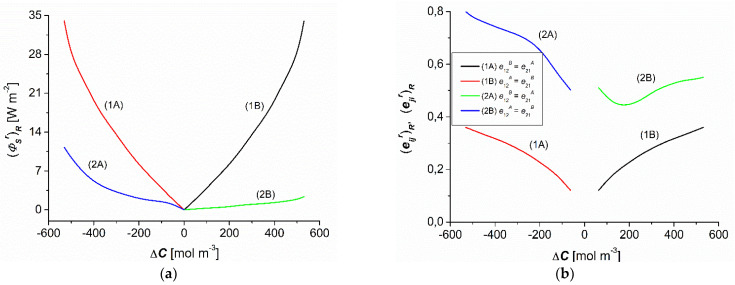
Concentration dependencies of ${\left(\Phi_{S}^{r}\right)}_{R}$ (**a**) and maximum energy conversion efficiency coefficients ${\left(e_{12}^{r}\right)}_{R}={\left(e_{21}^{r}\right)}_{R}$ (**b**) for aqueous ethanol solutions.

As shown in Figure 7a, the dependencies ${\left(\Phi_{S}^{r}\right)}_{R}$ = *f*(∆C) (*r* = A, B) calculated based on Equation (15) were nonlinear and symmetrical with respect the point Δ*C* = 0 for homogenous conditions (curves 1A and 1B), whereas they were complex and asymmetrical with respect to the vertical axis passing through the point ∆C = 0 for CP conditions (curves 2A and 2B). The comparison of curves 2A and 2B shows that for the conditions of concentration polarization and the same values (−∆C) and (∆C) the values of ${\left(\Phi_{S}^{A}\right)}_{R}$ (for the convective state) were greater than ${\left(\Phi_{S}^{B}\right)}_{R}$ (for the nonconvection state). On the other hand, when comparing curves 1A and 1B to curves 2A and 2B, it can be seen that for the same values of (−∆C) and (∆C) the values of ${\left(\Phi_{S}^{A}\right)}_{R}$ and ${\left(\Phi_{S}^{B}\right)}_{R}$ for homogeneous solution conditions were greater than for CP conditions. Compared to the conditions of homogeneity of solutions, for the same ∆C, CP reduced the value of the source of entropy ${\left(\Phi_{S}^{r}\right)}_{R}$. For the concentration polarization conditions, the values of ${\left(\Phi_{S}^{r}\right)}_{R}$ were positive and depended on both the value and the sign of ∆C. CP reduced the flux of dissipated energy (*S*-energy).

Curves 1A and 1B in Figure 7b show the dependencies ${\left(e_{ij}^{r}\right)}_{R}$ = *f*(∆C) for homogeneous conditions of solutions, calculated based on Equation (16). This figure shows that curves 1A and 1B are nonlinear and symmetrical with respect to the vertical axis passing through the point ∆C = 0. Curves 2A and 2B in Figure 7b show the dependencies of ${\left(e_{ij}^{r}\right)}_{R}$ = *f*(∆C) for CP conditions of the solutions. The figure shows that curves 2A and 2B are nonlinear complex curves that are asymmetric with respect to the vertical axis passing through the point ∆C = 0. Curves 1A and 1B show that for the conditions of homogeneity of solutions the condition was 0.12 ≤ ${\left(e_{12}\right)}_{R}$ = ${\left(e_{12}\right)}_{R}$ ≤ 0.36. On the other hand, curves 2A and 2B show that for the conditions of CP of solutions, the conditions were 0.5 ≤ ${\left(e_{12}^{A}\right)}_{R}$ = ${\left(e_{21}^{A}\right)}_{R}$ ≤ 0.8 and 0.44 ≤ ${\left(e_{12}^{B}\right)}_{R}$ = ${\left(e_{21}^{B}\right)}_{R}$ ≤ 0.55. Figure 6b also shows that ${\left(e_{12}^{A}\right)}_{R}$ = ${\left(e_{21}^{A}\right)}_{R}$ > ${\left(e_{12}^{B}\right)}_{R}$ = ${\left(e_{21}^{B}\right)}_{R}$ > ${\left(e_{12}\right)}_{R}$ = ${\left(e_{12}\right)}_{R}$. This indicates that the most intense energy conversion occurred in the A configuration for CP conditions. The values of the coefficients ${\left(e_{12}^{r}\right)}_{R}$ and ${\left(e_{21}^{r}\right)}_{R}$ increased in CP compared to the homogeneous conditions. For the conditions of CP, the values of these coefficients were positive and dependent on ∆C. Moreover, for the same ∆C the values of the coefficients ${\left(e_{12}^{r}\right)}_{R}$ and ${\left(e_{21}^{r}\right)}_{R}$ were greater for the convective state.

**Figure 8:**
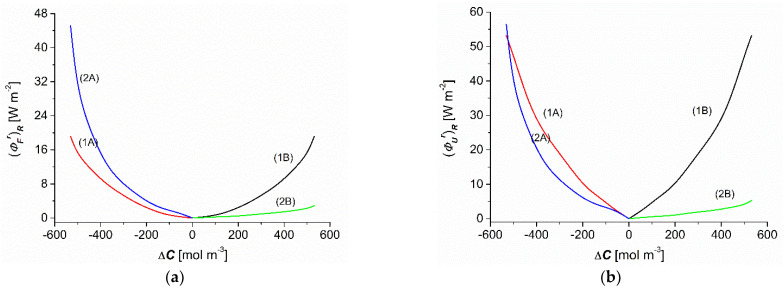
Concentration dependencies of the flux of *F*-energy ${\left(\Phi_{F}^{r}\right)}_{R}$ (**a**) and the flux of *U*-energy ${\left(\Phi_{U}^{r}\right)}_{R}$ (**b**) for aqueous ethanol solutions.

Taking into account the results of ${\left(\Phi_{S}^{r}\right)}_{R}$ and ${\left(e_{ij}^{r}\right)}_{R}$ shown in Figure 7a,b and Equations (19) and (20), the dependencies ${\left(\Phi_{F}^{r}\right)}_{R}$ = *f*(C) and ${\left(\Phi_{U}^{r}\right)}_{R}$ = *f*(∆C) were calculated. As shown in Figure 8a, the dependencies ${\left(\Phi_{F}^{r}\right)}_{R}$ = *f*(∆C) (*r* = A, B) calculated based on Equation (19) were nonlinear and symmetrical with respect to the point Δ*C* = 0 for homogenous conditions (curves 1A and 1B), whereas they were complex and asymmetrical with respect to the vertical axis passing through the point ∆C = 0 for CP conditions (curves 2A and 2B). The comparison of curves 2A and 2B shows that for the conditions of CP and the same values of (−∆C) and (∆C) the values of ${\left(\Phi_{F}^{A}\right)}_{R}$ (for the convective state) were greater than ${\left(\Phi_{F}^{B}\right)}_{R}$ (for nonconvection conditions). On the other hand, when comparing curves 1A and 1B to curves 2A and 2B, it can be seen that for the same values of (−∆C) and (∆C) the values of ${\left(\Phi_{F}^{A}\right)}_{R}$ and ${\left(\Phi_{F}^{B}\right)}_{R}$ for homogeneous solution conditions were greater than for CP conditions.

This tendency was maintained for dependence ${\left(\Phi_{U}^{r}\right)}_{R}$ = *f*(∆C). Figure 8a shows that the dependencies ${\left(\Phi_{U}^{r}\right)}_{R}$ = *f*(∆C), calculated based on Equation (20), were nonlinear and symmetrical with respect the point Δ*C* = 0 for homogenous conditions (curves 1A and 1B), whereas they were complex and asymmetrical with respect to the vertical axis passing through the point ∆C = 0 for CP conditions (curves 2A and 2B). The comparison of curves 2A and 2B shows that for the conditions of CP and the same values of (-∆C) and (∆C) the values of ${\left(\Phi_{U}^{A}\right)}_{R}$ (for the convective state) were greater than ${\left(\Phi_{U}^{B}\right)}_{R}$ (for nonconvection conditions). On the other hand, when comparing curves 1A and 1B to curves 2A and 2B, it can be seen that for the same values of (−∆C) and (∆C) the values of ${\left(\Phi_{U}^{A}\right)}_{R}$ and ${\left(\Phi_{U}^{B}\right)}_{R}$ for homogeneous solution conditions were greater than for CP conditions.

As shown in Figure 7a and Figure 8a,b, the following relations were satisfied for the homogeneity conditions of the solutions: ${\left(\Phi_{S}^{A}\right)}_{R}$ = ${\left(\Phi_{S}^{B}\right)}_{R}$ = ${\left(\Phi_{S}\right)}_{R}$, ${\left(\Phi_{F}^{A}\right)}_{R}$ = ${\left(\Phi_{F}^{B}\right)}_{R}$ = ${\left(\Phi_{F}\right)}_{R},$ and ${\left(\Phi_{U}^{A}\right)}_{R}$ = ${\left(\Phi_{U}^{B}\right)}_{R}$ = ${\left(\Phi_{U}\right)}_{R}$. In contrast, for the conditions of concentration polarization, the following relations were fulfilled: ${\left(\Phi_{S}^{A}\right)}_{R}$ > ${\left(\Phi_{S}^{B}\right)}_{R}$ < ${\left(\Phi_{S}\right)}_{R}$, ${\left(\Phi_{F}^{A}\right)}_{R}$ > ${\left(\Phi_{F}^{B}\right)}_{R}$, ${\left(\Phi_{F}^{A}\right)}_{R}$ > ${\left(\Phi_{F}\right)}_{R}$, ${\left(\Phi_{F}^{B}\right)}_{R}$ < ${\left(\Phi_{F}\right)}_{R}$, ${\left(\Phi_{U}^{A}\right)}_{R}$ > ${\left(\Phi_{U}^{B}\right)}_{R}$, ${\left(\Phi_{U}^{B}\right)}_{R}$ = ${\left(\Phi_{U}\right)}_{R}$ (for Δ*C* = −500 mol m^−3^), ${\left(\Phi_{U}^{B}\right)}_{R}$ < ${\left(\Phi_{U}\right)}_{R}$ (for Δ*C* > −500 mol m^−3^), ${\left(\Phi_{U}^{B}\right)}_{R}$ > ${\left(\Phi_{U}\right)}_{R}$ (for Δ*C* < −500 mol m^−3^), and ${\left(\Phi_{U}^{B}\right)}_{R}$ < ${\left(\Phi_{U}\right)}_{R}$.

The ${\left(\Phi_{F}^{r}\right)}_{R}$ is a measure of the flux of *F*-energy, that is, that part of the ($\Phi_{U}^{r}{)}_{R}$ that can be converted into useful work. The conversion efficiency of *U*-energy to *F*-energy for the same values of ∆C ranged from 12 to 36% (for conditions of homogeneity of solutions separated by the membrane). For diffusion conditions (configuration B), the efficiency of the *U*-energy to *F*-energy conversion was contained in the range from 50 to 57%, and for diffusion–convection conditions (configuration B) the efficiency ranged from 50 to 79%. Although this efficiency was relatively high, the amount of *F*-energy produced was small. The amount of this energy can be important in biological microsystems.

The procedure presented in this paper for evaluating membrane transport properties and energy conversion in a membrane system could be useful for any biological or artificial membranes [46,47,48,49,50].

## 4. Conclusions

This study presents the following results:

- Developed within the framework of the Kedem–Katchalsky–Peusner formalism, the procedure using the Peusner coefficients $R_{ij}^{r}$ (*i* = *j* ∈ {1, 2}, *r* = A, B) and $R_{det}^{r}$ is suitable for evaluating the transport properties of polymer membranes and assessing the conversion of internal energy (*U*-energy) to useful energy (*F*-energy) and degraded energy (*S*-energy).
- Peusner coefficients $R_{12}^{r}$ and $R_{21}^{r}$ are related to the membrane Peclét coefficients $\alpha_{s}^{r}$ and $\alpha_{v}^{r}$.
- The procedure developed in this paper to evaluate the conversion of internal energy (*U*-energy) to useful energy (*F*-energy) and degraded energy (*S*-energy) requires the calculation of the value of the flux of *S*-energy ${\left(\Phi_{S}^{r}\right)}_{R}$ and efficiency factors ${\left(e_{12}^{r}\right)}_{R}$ and ${\left(e_{21}^{r}\right)}_{R}$, followed by the fluxes of *F*-energy $(\left(\Phi_{F}^{r}{)}_{R}\right)$ and *U*-energy (${\left(\Phi_{U}^{r}\right)}_{R}$).
- The procedure proposed in the paper can be applied to membranes for which the coefficients $L_{P}$, $\sigma_{v}$, $\sigma_{s}$, and ω can be determined experimentally.

## Data Availability

The datasets for this study are available on request from the corresponding author.

## List of Symbols

$L_{P}$	hydraulic permeability coefficient (m^3^N^−1^s^−1^)
$\bar{C}$	average concentration of solutes (mol m^−3^)
$\zeta_{p}^{r}$ $,\;\zeta_{v}^{r}$ $,\;\zeta_{s}^{r}$ $,\;\mathrm{and}\;\zeta_{a}^{r}$	hydraulic, osmotic, diffusive, and advective coefficients of CP
$R_{ij}^{r}$$\;\mathrm{and}\;R_{det}^{r};$ i, j ∈ {1, 2}, r = A, B	$\mathrm{Peusner}\;\mathrm{coefficients}\;(R_{11}^{r}$$\;(N\;s\;m^{-3}),\;R_{12}^{r}$$\;(N\;s\;{\mathrm{mol}}^{-1}),\;R_{21}^{r}$$\;(N\;s\;{\mathrm{mol}}^{-1}),\;\mathrm{and}\;R_{det}^{r}$ (N^2^s^2^mol^−2^))
$\delta_{h}^{r}$ $\;\mathrm{and}\;\delta_{l}^{r}$	thicknesses of the concentration boundary layers (CBLs) (m)
$e_{ij}^{r}$	energy conversion efficiency coefficients
$R^{r}$	matrix of the Peusner coefficients
$\Phi_{S}^{r}$	flux of S-energy (W m^−2^)
$\Phi_{F}^{r}$	flux of F-energy (W m^−2^)
$\Phi_{U}^{r}$	flux of U-energy (W m^−2^)
$\rho_{h}$ $\;\mathrm{and}\;\rho_{l}$	mass density (kg m^−3^)
$r_{ij}^{r}$	coupling coefficient
$R_{C}$	concentration Rayleigh number
${\left(\phi_{ij}^{r}\right)}_{R}$ $\;\mathrm{and}\;{\left(\phi_{det}^{r}\right)}_{R}$	concentration polarization effects
${\left(\varphi_{ij}\right)}_{R}$ $\;\mathrm{and}\;{\left(\varphi_{det}\right)}_{R}$	effects of gravitational convection in osmotic and diffusive transport
$P_{e}$	Peclét number
$\alpha_{s}^{r}$ $\;\mathrm{and}\;\alpha_{v}^{r}$	$\mathrm{Pecl}\text{é}t\;\mathrm{coefficients}\;(\alpha_{s}^{r}$$\;(m^{2}s\;{\mathrm{mol}}^{-1})\;\mathrm{and}\;\alpha_{v}^{r}$ (s m^−1^))
${℘}_{v}$ $\;\mathrm{and}\;{℘}_{s}$	$\mathrm{solute}\;\mathrm{permeability}\;\mathrm{coefficient}\;({℘}_{v}$$\;(m\;s^{-1})\;\mathrm{and}\;{℘}_{s}$(mol m^−2^s^−1^))
A and B	configurations of membrane system
M	membrane
CP	concentration polarization
BC	bacterial cellulose
$l_{l}^{r}$ $\;\mathrm{and}\;l_{h}^{r}$	the concentration boundary layers (CBLs)
$l_{h}^{r}$ $/M/l_{l}^{r}$	complex of CBLs and membrane
KKP equations	Kedem–Katchalsky–Peusner equations
